# Graph neural networks for integrated information and major complex estimation

**DOI:** 10.1371/journal.pone.0335966

**Published:** 2025-11-07

**Authors:** Tadaaki Hosaka

**Affiliations:** School of Science and Technology, Meiji University, Kanagawa, Japan; Central South University, CHINA

## Abstract

This study investigates the potential of graph neural networks (GNNs) for estimating system-level integrated information and major complex in integrated information theory (IIT) 3.0. Owing to the hierarchical complexity of IIT 3.0, calculating the integrated information and identifying the major complex are computationally prohibitive for large systems. To overcome this difficulty, we propose a GNN model with transformer convolutions characterized by multi-head attention mechanisms for estimating the major complex and its integrated information. For evaluation, we begin by obtaining exact solutions for integrated information and major complexes in systems with 5, 6, and 7 nodes, and conduct two experiments: (1) a non-extrapolative setting in which the model is trained and tested on a mixture of systems with 5, 6, and 7 nodes, and (2) an extrapolative setting in which systems with 5 and 6 nodes are used for training and systems with 7 nodes are used for testing. We then examine the scaling behavior for tree-like, fully connected, and loop-containing graph topologies in larger systems. Although accurate estimation is difficult, our approximate estimates for larger systems generally preserve the qualitative patterns of integrated information and major complex size that are observed in small systems. Finally, based on this observation, we qualitatively analyze a split-brain–like system of 100 nodes. The system consists of two weakly coupled subsystems of 50 nodes each, representing a structurally meaningful, brain-inspired configuration. When the connectivity between the subsystems is low, “local integration” emerges, and a single subsystem forms a major complex. As the connectivity increases, local integration rapidly disappears, and the integrated information gradually rises toward “global integration,” in which a large portion of the entire system forms a major complex. Our analysis suggests that the proposed GNN-based framework provides a practical approach to qualitative analysis of integrated information and major complexes in large systems.

## Introduction

Integrated information theory (IIT), proposed by Giulio Tononi, has witnessed significant development over the last two decades, evolving through four major versions [1–7] and becoming a central framework for understanding consciousness. IIT asserts that consciousness emerging in a system is equivalent to the integrated information quantified by Φ, which measures the information in the whole exceeding the sum of the information in its parts. This core principle is a fundamental concept shared across all versions of IIT.

IIT 1.0 [1] introduced the foundational notion that consciousness arises from the integrated information within a system, emphasizing the difference between the whole and its parts. In IIT 2.0 [2–4], this idea was rigorously formalized through the introduction of the “minimum information partition.” It is defined as the division that yields the smallest decrease in information integration relative to the non-partitioned system. However, IIT 2.0 treated the entire system as a single entity for calculating the integrated information without considering that consciousness might be composed of integrated subsystems or exploring any hierarchical structures within the system. By contrast, IIT 3.0 [5,6] introduced a hierarchical approach that analyzes all possible subsystems to elaborate the nature of consciousness more precisely (S1 Text). For every group of nodes within each subsystem, the mechanism-level integrated information, denoted as φ, is measured according to the principle of the minimum information partition. From the collection of these values of φ and their associated probability distributions, the system-level integrated information for each subsystem is then computed as Φ. Finally, the subsystem with the highest Φ across the entire system is identified as the “major complex,” which represents the location of consciousness within the system. The latest version, IIT 4.0 [7], has advanced the theory by introducing the concept of “relations” between node groups to refine how the value of Φ is derived from the collection of φ.

This study is based on IIT 3.0. For a system characterized by nodes, edges, node states, and state transition probabilities, the primary objective of IIT 3.0 is to identify the “major complex,” defined as the subset of nodes with the highest integrated information Φ. The most notable advancement from IIT 2.0 to IIT 3.0 is the introduction of the aforementioned hierarchical structure to compute the system-level integrated information, which significantly increases the computational complexity owing to more intricate hierarchical procedures. Deriving integrated information Φ and major complex pose substantial computational challenges, not only because finding the minimum information partition requires evaluating all possible partitions of a target system but also because IIT 3.0 involves multiple nested combinatorial optimization problems, as described in S1 Text. Consequently, the exponential growth in computational complexity with the number of nodes restricts rigorous IIT 3.0 calculations to extremely small systems, typically those comprising fewer than 10 nodes, thereby limiting the applicability of IIT 3.0 to large-scale systems.

Several theoretical and numerical studies have explored the specific characteristics and limitations of IIT 3.0 [8–11], but practical approximation methods have not been proposed thus far. Although efficient approximation methods for identifying optimal partitioning patterns have been proposed in the context of IIT 2.0 [12,13], they cannot be applied to IIT 3.0 due to its complex hierarchical structure. An attempt within the IIT 3.0 framework is the “cut-one” approximation [14], which restricts the search space for the system-level partition to those that isolate a single node. However, this method simplifies only one of the multiple combinatorial optimizations required by IIT 3.0, and the overall computational complexity remains exponential with respect to the number of nodes. To date, no practical approximation method capable of fully bypassing the computational difficulties of IIT 3.0 has been proposed.

To extend the applicability of IIT to larger systems, it is crucial to address its computational challenges. Graph neural networks (GNNs) offer a promising solution to this problem. GNNs are a type of deep learning model designed specifically for graph-structured data, where nodes represent entities and edges represent their relationships. GNNs have been effectively employed in various biological applications, including drug discovery [15], predicting protein interfaces [16,17], modeling neural connectivity patterns in the brain [18], identifying relationships between diseases and genes [19], and classifying lung cancer subtypes from whole-slide images [20]. They have also been applied in other fields such as predicting structural dynamic responses in civil engineering [21], state estimation in power systems [22], weather forecasting [23], recommending personalized items based on user behaviors [24], and analyzing train-bridge coupled systems [25,26]. Although GNNs face challenges in terms of interpretability, they often outperform traditional machine learning methods by accurately extracting complex patterns from data. Unlike traditional feature-based machine learning models such as support vector machines or multilayer perceptrons, GNNs can directly leverage the topological structure of the input graph. This capability is essential for tasks that depend on the graph topology and inter-node relationships. Such a property could make GNNs particularly suitable for estimating integrated information and major complex in IIT 3.0, without explicitly modeling the intricate computational processes.

Isomorphism invariance means that the output does not depend on how node labels are assigned but only on the underlying graph structure. This property is essential for GNNs to estimate integrated information Φ and the major complex, since both are determined solely by the structural and probabilistic properties of the system. Most existing GNNs operate as message-passing algorithms [27], where each node iteratively aggregates information from its neighbors to update its feature representation. This process is often referred to as graph convolution, and its expressive power is theoretically equivalent to that of the first-order Weisfeiler–Lehman (1-WL) graph isomorphism test [28,29]. Consequently, graph convolutional networks may fail to distinguish between non-isomorphic graphs.

To overcome the expressiveness limitations of 1-WL-based models, higher-order extensions such as *k*-WL tests and their corresponding *k*-GNN architectures have been proposed [30]. These models consider combinations of *k* nodes rather than individual nodes. With a sufficiently large value of *k* and appropriate parameters, they can theoretically approximate any continuous graph function [31]. However, their computational and memory demands grow rapidly with the number of nodes, making them impractical for large-scale systems. As a more practical alternative, efforts have been made to enhance the performance of 1-WL-equivalent models. For example, the Graph Isomorphism Network [29] improves expressiveness through careful aggregation design, while transformer-based GNNs [32] incorporate attention mechanisms inspired by advances in natural language processing [33]. These approaches aim to maximize the capability of 1-WL models while maintaining computational feasibility, aligning well with the goals of our study. In particular, we adopt transformer convolution in our framework to leverage its expressive power and scalability.

Our GNN-based framework does not attempt to model explicitly the complex nested optimization procedures of IIT 3.0. Instead, it adopts a data-driven approach that aims to learn the mapping from system structure to the values of Φ and the major complex. This means that the model is designed to imitate only the input–output relationship derived from IIT calculations. We consider this approach a meaningful and practical strategy for extending IIT to large systems where exact computation is infeasible.

This study investigates the potential of GNNs as an approximation method for estimating the major complex and its maximum integrated information Φ. We first evaluate the performance of GNNs on small-scale systems where exact theoretical calculations are feasible. After confirming the persistence of qualitative trends in Φ and major complex size from small to larger systems, we qualitatively investigate large systems that resemble a split-brain scenario [34,35]. This allows us to assess the applicability of our approach to structurally meaningful, brain-inspired configurations. Through these experiments, we show that the proposed framework can capture qualitative patterns of integrated information and major complex formation in large systems. Hereafter, the term “integrated information” and the variable Φ will refer to the maximum value of the system-level integrated information across all subsystems, i.e., the integrated information of the major complex, unless otherwise noted.

While the present study adopts the framework of IIT 3.0, this choice does not imply a limitation of scope. IIT 4.0 can be viewed as an incremental refinement that builds upon the foundation of IIT 3.0, and both versions share the same objective: computing the system-level integrated information Φ and identifying the major complex. Our proposed GNN-based framework predicts these quantities directly from graph features, without relying on intermediate constructs specific to any particular version of the theory. In this sense, our method is compatible with IIT 4.0.

## Methods

In this study, we estimate integrated information Φ and major complex for randomly generated systems consisting of N(=5,6,7) nodes. This limitation on the number of nodes is necessary because obtaining the exact solutions for IIT 3.0 becomes computationally infeasible for larger systems. For each value of *N*, numerous random systems are generated and used to train the GNN model.

Each node in the system can be in one of two states, +1 or –1, denoted as *S*_*i*_ (i=1,2,…,N), and the states are randomly assigned. The undirected edges between any pair of nodes are assigned with a probability of *p* = 0.4, and the edge weights *J*_*ij*_ between nodes *i* and *j* are randomly determined following a standard normal distribution. The conditional probability $p_{i}(s|S)$ that node *i* will take on state s∈{1,−1} in the next time step is determined on the basis of the current states $S=(S_{1},S_{2},\ldots ,S_{N})$ using the Boltzmann distribution:

$$p_{i}(s|S)=\frac{1}{1+\exp\left(-\frac{2\tau_{i}s}{T}\right)},$$ (1)

where $\tau_{i}=\sum_{j\in 𝒩(i)}J_{ij}S_{j}$ represents the input to node *i*, and 𝒩(i) denotes the set of nodes connected with node *i*. The parameter *T*, controlling the sharpness of the distribution, is uniformly sampled from the range [0.1,3.0] and is set independently for each generated system. This stochastic model captures the dynamics of the system, allowing probabilistic determination of node states based on neighborhood interactions.

Using the PyPhi library published by Mayner et al. [14], the exact solutions for the integrated information Φ and the corresponding major complex are obtained for each of the randomly generated systems. These systems and their solutions serve as the dataset for subsequent analysis within a supervised learning framework, where a GNN is trained on part of the dataset and tested on the remaining data.

Each system is treated as a graph where nodes and edges have specific features. Node i (i=1,2,…,N) is assigned a feature vector consisting of the following six dimensions:

1. $\max\{p_{i}(1|S),p_{i}(-1|S)\}$: The larger value between the probability that node *i* will be in state +1 in the next time step and the probability that it will be in state –1. Note that the node states S affect only this feature and have no influence on the other features.
2. Parameter *T*: A parameter that controls the sharpness of the probability distribution. In each graph, all nodes share the same value of *T*.
3. Node degree: The number of edges connected to node *i*, indicating its connectivity in the system.
4. Closeness centrality: A measure of how close node *i* is to all other nodes in the system, calculated as the inverse of the average of the shortest path distances from node *i* to all other nodes. It indicates how rapidly information spreads from node *i* to others.
5. Betweenness centrality: A measure of how frequently node *i* lies on the shortest paths between pairs of other nodes. It indicates the importance of node *i* in the network communication.
6. Clustering coefficient: A measure of how closely the neighbors of node *i* are connected to each other, indicating the density of local connections around node *i*. It is calculated as the ratio of actual connections to possible connections among the neighbors of node *i*.

Meanwhile, the edge features simply consist of a single dimension representing the edge weights, *J*_*ij*_, between the nodes.

It should be noted that the node state *S*_*i*_ is not utilized as a feature. The random systems in our study exhibit invariance under inversion of the state vector. Specifically, the conditional probabilities of transitions are symmetric, indicating that $p(S^{*}|S)=p(-S^{*}|$ − S), where $S^{*}$ denotes a specific state to which the system transitions from the current state S. These conditional probabilities are derived by the product of Eq 1 over all the nodes, assuming conditional independence. According to IIT 3.0, for such symmetrical systems, both the state vector S and its inverse −S should yield the same integrated information and major complex. However, if the node state *S*_*i*_ is included as a node feature in the GNN, the use of ReLU activations and bias terms could violate this invariance, leading to different outputs for S and −S despite their theoretical equivalence. We have designed the six aforementioned features to be identical for both node states S and −S, ensuring that the GNN produces theoretically consistent predictions.

**Fig 1:**
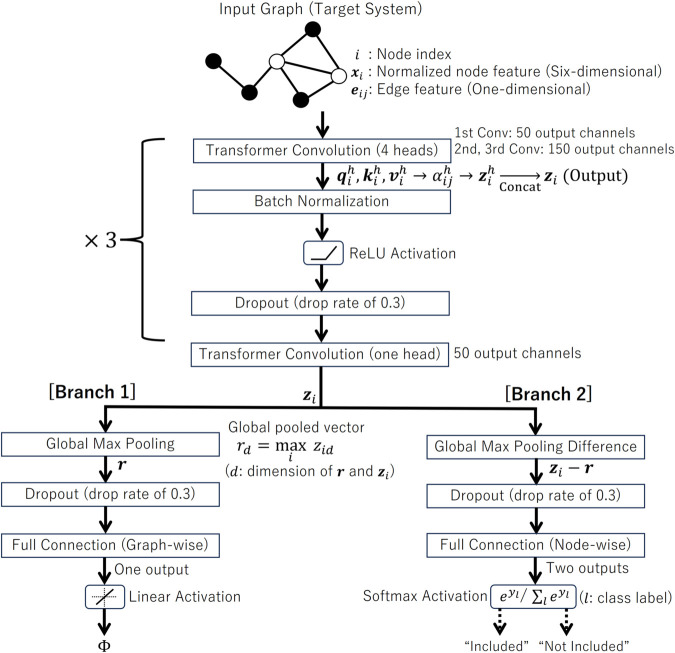
Architecture of the GNN used to estimate the integrated information and the major complex. The input node features are processed through four consecutive transformer convolutional layers with multi-head attention. After the fourth layer, the network splits into two branches to realize multi-task learning. One branch performs graph-level pooling followed by a fully connected layer to estimate the system-level Φ, while the other branch compares pooled and node-level features to classify whether each node belongs to the major complex.

Fig 1 shows our GNN architecture, which is designed to perform multi-task estimation, i.e., predicting the integrated information Φ and identifying the major complex for a target system. The input consists of the six-dimensional feature vector for every node, with each dimension normalized using the entire training dataset. These inputs pass through four consecutive transformer convolutional layers. In each layer, attention weights are dynamically adjusted on the basis of the relationships between nodes [32], which has been highly successful in natural language processing [33]. Batch normalization and dropout follow each of the first three transformer layers to stabilize training and prevent overfitting.

Our model employs the multi-head attention mechanism, where each head is expected to independently capture different aspects of the relationships between nodes. Specifically, *d*-dimensional feature vector of each node, $x_{i}\in {\mathbb{R}}^{d}$, is transformed into three components, namely query ($q_{i}$), key ($k_{i}$), and value ($v_{i}$), through learnable weight matrices $W_{q}$, $W_{k}$, and $W_{v}$. The transformations for each head h(=1,2,…,H) are given by

$$q_{i}^{h}=W_{q}^{h}x_{i},\;k_{i}^{h}=W_{k}^{h}x_{i},\;v_{i}^{h}=W_{v}^{h}x_{i},$$ (2)

where $W_{q}^{h}$, $W_{k}^{h}$, and $W_{v}^{h}$ are in ${\mathbb{R}}^{C\times d}$ and *C* represents the number of output channels. The attention weight $\alpha_{ij}^{h}$ toward node *i* from its neighbor *j* is computed using the dot product of the query from node *i* and the key from node *j*, with the addition of a term based on the edge feature $e_{ij}$ (one dimension in our study) and its learnable transformation matrix $W_{e}^{h}$:

$$\alpha_{ij}^{h}=\text{softmax}\left(\frac{q_{i}^{h}\cdot (k_{j}^{h}+W_{e}^{h}e_{ij})}{\sqrt{\kappa }}\right).$$ (3)

Here, $\alpha_{ij}^{h}$ represents the importance of the feature of node *j* for updating node *i*, and κ is a scaling parameter typically set to *C*/*H*. The softmax operation ensures that the incoming attention weights to each node are normalized so that their sum is 1. The updated feature for node *i* in each head is then computed as a weighted sum of the value vectors from its neighboring nodes:

$$z_{i}^{h}=W_{0}^{h}x_{i}+\sum_{j\in 𝒩(i)}\alpha_{ij}^{h}\left(v_{j}^{h}+W_{e}^{h}e_{ij}\right),$$ (4)

where matrix $W_{0}^{h}$ is in ${\mathbb{R}}^{C\times d}$. The outputs from the multiple heads are concatenated to form the final updated feature for node *i*:

$$z_{i}=\text{Concat}(z_{i}^{1},z_{i}^{2},\ldots ,z_{i}^{H}),$$ (5)

which results in a feature vector $z_{i}$ of dimension *CH*. This multi-head attention mechanism enables our GNN to attend to different aspects of the input graph simultaneously.

Following the fourth transformer layer, the network splits into two separate branches. In the first branch, the graph-level features r are obtained using global max pooling, which selects, for each feature dimension, the maximum value across all nodes in the graph. Then, this pooled feature vector is passed through a dropout layer, followed by a fully connected layer that outputs the estimated integrated information Φ for the entire system.

In the second branch, the global max-pooled vector r is subtracted from the feature vector of each node. This operation highlights the relative importance of each node compared to the most prominent features across the graph. Subsequently, the resulting differences are passed through a fully connected layer with a softmax activation function to perform a binary classification, determining whether each node is included in the major complex.

**Fig 2:**
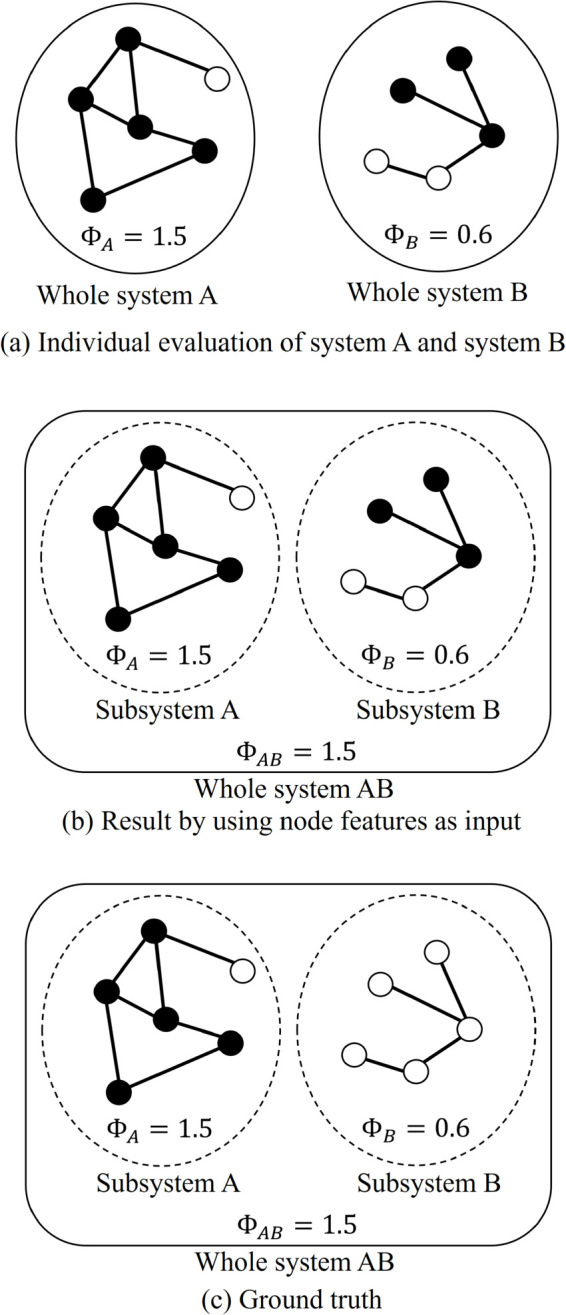
Integrated information Φ and major complex in a system consisting of two disconnected subsystems. (a) When each system is evaluated individually, the integrated information and the major complex can be correctly estimated using node features alone as input to the fully connected layer, without subtracting the max-pooled vector. Black circles represent nodes included in the major complex, while white circles represent nodes not included. (b) Since no edge exists between the two subsystems, the results of the transformer convolutions are identical to those obtained when each subsystem is evaluated independently. However, this leads to an incorrect estimation. (c) In the ground truth, the nodes in the subsystem with the smaller Φ should not be included in the major complex.

The subtraction of the global max-pooled vector from the feature vector of each node is crucial for improving the classification performance. Using only individual node features as inputs to the fully connected layer in Branch 2 can cause significant errors when the target system consists of multiple disconnected subsystems. As illustrated in Fig 2(a), when system A or system B alone constitutes the whole system, we assume that the GNN accurately predicts the integrated information $\left(\Phi_{A}>\Phi_{B}\right)$ and identifies the major complex for each subsystem. Even under this assumption, the prediction fails when subsystems A and B are evaluated together as a single disconnected larger system (Fig 2(b), 2(c)). In this larger system, the graph convolution results for subsystems A and B remain identical to those obtained when the subsystems are evaluated individually. As a result, the GNN misclassifies some nodes from the subsystem with lower integrated information (B in this case) as belonging to the major complex. This misclassification occurs because using only the individual node features without considering the global structure fails to differentiate between the subsystems. Subtracting the global max-pooled vector from each node feature allows the model to capture relative importance. Consequently, nodes from the subsystem with the highest integrated information (A in this case) are more likely to be correctly identified as part of the major complex.

## Results

In this section, we present the results of our simulations. We employed the PyTorch Geometric library [36] to implement the GNNs in the following experiments. Computations were mainly performed on a workstation equipped with an Intel Core i9 2.00 GHz processor, 128 GB of RAM, and an NVIDIA RTX A4500 GPU (20 GB memory). To evaluate the performance of our GNN model in estimating integrated information Φ and classifying the inclusion of the nodes in the major complex, we conducted three types of experiments.

The first experiment used a dataset of 3000 graphs in total, consisting of 1000 random systems generated as described in the previous section for each case of N=5,6, and 7. The entire dataset was randomly shuffled and then split into two subsets: 90% of the total data was used in the training process, while the remaining 10% was reserved for testing. Then, the performance of the model was evaluated on the test dataset by measuring the accuracy of both the integrated information estimation and the classification of whether each node belongs to the major complex.

The second experiment aimed to assess the ability of the model to generalize to unseen system sizes slightly larger than those used in training, which was regarded as an extrapolative setting. We trained the model using a dataset consisting of 1500 graphs for each case of *N* = 5 and 6, i.e., a total of 3000 random systems. The test dataset consisted of 1000 random systems for *N* = 7. The performance of the model was evaluated on this test dataset with the same metrics as in the first experiment.

Finally, using the GNN models trained in the first experiment, we extended the evaluation to larger systems. This included an examination of scaling behavior across representative graph topologies and a qualitative test on a structured large-scale example. In the latter, we designed a test system inspired by the split-brain scenario [34,35]. The system comprises two subsystems, each of which contains 50 nodes. By varying the probability of an edge connecting nodes in different subsystems, we investigated changes in the estimated integrated information and major complex, highlighting the transition from local to global integration.

For the three types of experiments, we applied several common settings and parameters that were empirically determined. We defined two class labels: label *in*, representing nodes “included in the major complex,” and label *out*, representing nodes “not included in the major complex.” To address the imbalance between the numbers of these classes, meaning that label *in* was more frequent in our dataset, we applied a penalty factor of 1.8 to label *out* in the cross-entropy loss calculation. The total loss function for our multi-task model consisted of the sum of the mean squared error for the integrated information estimation and five times the cross-entropy loss for the major complex classification. This weighting factor of five was empirically introduced to balance the two tasks. The optimizer was set to LION (evolved sign momentum) [37]. This optimizer updates parameters based only on the gradient sign, whereas traditional optimizers such as SGD and Adam [38] also use the gradient magnitude. The LION optimizer provides a simple yet effective optimization strategy, focusing on efficiency and memory-saving features suitable for training deep network models. The learning rate in the algorithm was set to 0.0001 and the mini-batch size was set to 128.

Furthermore, to address the imbalance between label *in* and label *out* in the training dataset, we adopted a data augmentation strategy to artificially increase the number of label *out* instances. Specifically, we generated additional systems amounting to 5% of the total data used in the training process. To do this, we randomly selected two graphs from the training dataset and treated them as disconnected subsystems within a larger system. Assuming that the integrated information values of the two selected graphs are $\Phi_{A}$ and $\Phi_{B}$ with $\Phi_{A}>\Phi_{B}$ as in Fig 2(a), the whole of this artificially created system would have an integrated information value of $\Phi_{A}$. The true label for major complex inclusion would be retained for nodes from graph A, while all nodes from graph B would be assigned the label *out* as in Fig 2(c), thereby increasing the proportion of *out* labels in the dataset.

As the distribution of Φ values within the training dataset was not uniform, an oversampling technique was adopted to improve the estimation accuracy in the low-frequency regions. We used k-means clustering to divide the data into seven bins based on the value of Φ. Then, we oversampled the smaller bins to match the size of the largest bin, adding noise by applying a multiplicative factor of 5% independently to both the integrated information values Φ and each dimension of the node features.

Following the application of data augmentation and oversampling techniques, 10% of the expanded training dataset was set aside as validation data to monitor the performance of the model during training. The training process was halted if the validation loss did not improve for 50 consecutive epochs, following the early stopping strategy.

### Estimation in non-extrapolative setting

The results of the first experiment are summarized in Table 1. The table includes four main metrics evaluated on the test data: the mean squared error (MSE) and correlation coefficient between the estimated and actual values of the integrated information Φ, the accuracy of the node-wise prediction for inclusion in the major complex, and the graph-wise accuracy, i.e., the proportion of graphs where the major complex is predicted completely correctly. The values shown in the table are averages obtained over 100 repetitions of the experiment, where different combinations of training and testing datasets were used. The table presents the performance achieved with the proposed method, and for comparison, it also shows the following:

- Performance when removing each dimension of the node features.
- Performance when replacing the transformer convolutional layer with more standard convolutional types, specifically the graph convolutional layer (GraphConv class provided in the PyTorch Geometric library) and graph attention network [39] (GAT class in the PyTorch Geometric library). GraphConv simply aggregates features from neighboring nodes using a weighted sum without attention mechanisms. GAT employs simpler attention mechanisms than the transformer convolution.
- Performance when replacing LION with other optimizers, specifically Adam [38], which is widely regarded as the de facto standard, and its derivatives RAdam [40] and AdamW [41].
- Performance for single-task scenarios, where either integrated information Φ or major complex is estimated independently. This corresponds to evaluations on networks with either Branch 1 or Branch 2 removed (see Fig 1).
- Performance under two pooling settings:
  1. using global average pooling instead of global max pooling in Branches 1 and 2,
  2. using node features as inputs to the fully connected layer in Branch 2 without subtracting the max-pooled vectors.

**Table 1:** Performance evaluation with consistent training and testing node sizes (non-extrapolative). The performance of the proposed method is compared against variations in node features, convolution types, optimizers, and pooling strategies, as well as single-task estimations. The numbers in parentheses for the proposed method indicate its rank in each column, and results outperforming the proposed method are underlined.

Category	Configuration	Integrated information estimation		Major complex estimation	
		MSE	Correlation	Node-wise accuracy	Graph-wise accuracy
Proposed method		0.4611 (2)	0.7446 (2)	0.8574 (2)	0.5779 (3)
Removed feature	Probability	0.5272	0.7061	0.8544	0.5615
	Parameter *T*	0.5128	0.7149	0.8492	0.5566
	Node degree	0.5122	0.7076	0.8554	0.5727
	Closeness centrality	0.5585	0.6870	0.8521	0.5663
	Betweenness centrality	0.4920	0.7206	0.8550	0.5680
	Clustering coefficient	0.5362	0.7061	0.8443	0.5437
Substitute convolution	GraphConv	0.6254	0.6799	0.8107	0.4650
	GAT	0.5706	0.6950	0.7865	0.4168
Substitute optimizer	Adam	0.4828	0.7331	0.8572	0.5780
	RAdam	0.4899	0.7364	0.8589	0.5793
	AdamW	0.4780	0.7413	0.8565	0.5742
Single task	Φ estimation	0.4638	0.7376	-	-
	Major complex estimation	-	-	0.8557	0.5755
Pooling	Global average	0.4712	0.7380	0.8397	0.5207
	Not subtraction	0.4495	0.7547	0.8281	0.4908

**Fig 3:**
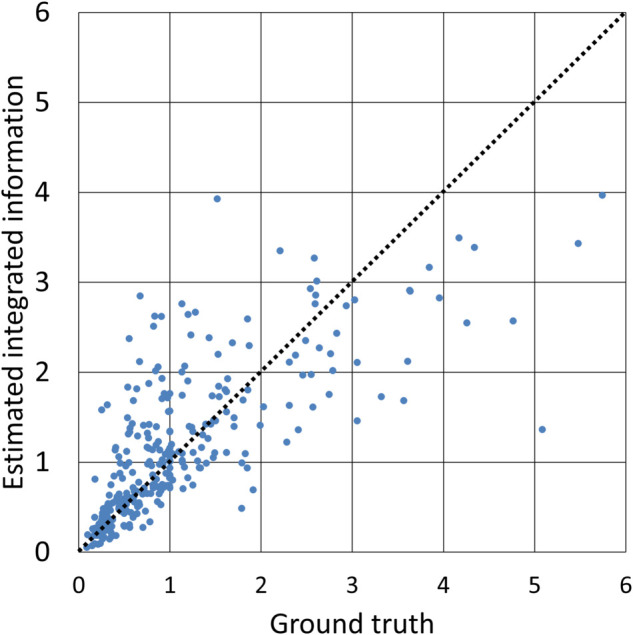
Scatter plot between estimated and true integrated information (non-extrapolative). All 300 test data used in one of the 100 trials conducted with the proposed method are shown. In this case, the correlation coefficient was 0.7464, which was close to the average over all the trials. Accurately estimated instances are located on the dotted line with a slope of 1.

The performance of the proposed method in estimating integrated information shows a relatively strong correlation coefficient of 0.7446 between the estimated and true values, implying a reasonable level of predictive capability. Fig 3 shows a scatter plot comparing the estimated and actual values of Φ in one trial. Most systems had the values of Φ below 1, where the model demonstrated higher accuracy. By contrast, systems with Φ values around 1 or higher were less frequent and tended to be predicted with lower accuracy. This uneven distribution with respect to the values of Φ led to greater variability in the higher-Φ regions. While oversampling could increase the apparent number of samples, it did not sufficiently reproduce the diverse characteristics of graph structures and node features in regions with few samples, which might limit its effectiveness in improving prediction accuracy. This result suggests that having a more balanced set of real samples could help improve the model’s robustness across the entire range of Φ values.

When predicting node inclusion in the major complex, the proposed method achieves a node-wise accuracy of 0.8574, indicating reliable performance in this binary classification task. The graph-wise accuracy measures the proportion of graphs in which the major complex is completely identified, and it is 0.5779 in this experiment. Interestingly, this is higher than the expected value obtained by simply raising the node-wise accuracy to the power of the number of nodes (5, 6, or 7). This suggests that our model captures a certain degree of interdependence among nodes forming the major complex, rather than treating each node independently. Specifically, the model seems to learn characteristic patterns in the graph structure associated with major complex, and to make incorrect predictions on graphs that deviate from these learned patterns. Further improvements can be achieved by intensively training on rare graph configurations.

When a single node feature is removed, the decline in accuracy for major complex estimation is relatively small. By contrast, the performance related to Φ estimation shows a more pronounced decrease, indicating that integrated information is more sensitive to changes in node features. Since both the probability of a node being in state +1 or –1 and the parameter *T* are closely related to the state transition probabilities required for the IIT calculation, it is intuitively expected that they would have a larger impact than the centrality-based features. However, we do not find significant differences to identify the best-performing feature.

Meanwhile, the selection of convolution type has a greater influence on performance compared to node feature reduction. The standard convolutional methods, GraphConv and GAT, resulted in much lower performance compared to our transformer-based approach. This result highlights the effectiveness of the weight determination of the attention mechanism in the transformer, which has also proven successful in natural language processing.

In addition, although the Adam and the RAdam optimizers slightly outperform the proposed method in terms of major complex estimation accuracy, the overall performance of LION, Adam, RAdam, and AdamW shows no significant differences. However, the proposed method using LION demonstrates a clear advantage in efficiency, with an average convergence time of 94.2 epochs, compared to 166.2 epochs for Adam, 185.4 epochs for RAdam, and 160.8 epochs for AdamW. This highlights the effectiveness of the LION optimizer, designed primarily to enhance efficiency rather than accuracy.

Estimating integrated information and major complex with the multi-task approach resulted in limited performance improvements compared to single-task models. This suggests that, although deriving integrated information and identifying major complex are theoretically related within the framework of IIT 3.0, the proposed method does not fully capture their deeper interconnections. Enhancing the ability of the multi-task model to better utilize the intricate relationships between integrated information and major complex is a key challenge for future work.

Using global average pooling fails to achieve the same level of accuracy as global max pooling, especially for major complex estimation, even though average pooling is commonly used for graph-wise prediction. This highlights the importance of global max pooling in the proposed method. Global max pooling emphasizes the most dominant feature signals by taking the element-wise maximum across all nodes. Average pooling, however, tends to dilute such signals, leading to reduced accuracy. Similarly, using node features directly as inputs to the fully connected layer in Branch 2 (see Fig 1), without subtracting the global max-pooled vector, significantly degrades the major complex estimation accuracy. Although this approach slightly improves estimation accuracy of Φ, subtracting the max-pooled vector offers a distinct theoretical advantage for major complex estimation, which outweighs those small gains.

**Fig 4:**
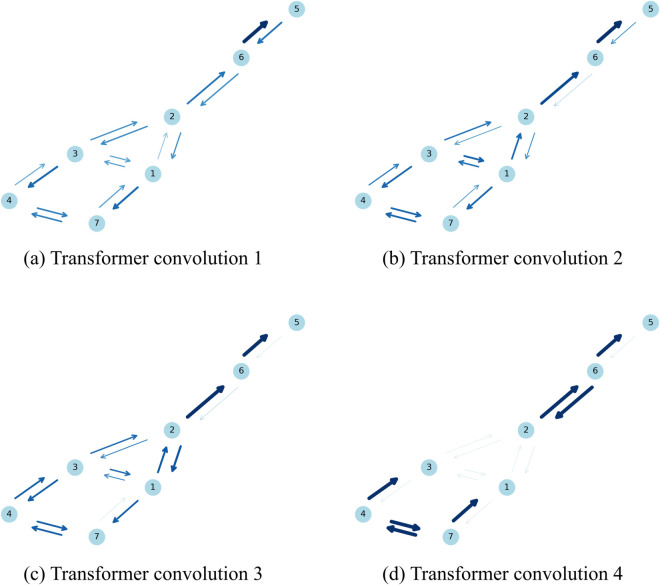
An example of attention weights visualization. This figure shows the weights between neighboring nodes at each transformer layer for a system with *N* = 7 nodes and Φ=8.036. Edge thickness and color intensity indicate the magnitude of the attention weights. Except for the last transformer layer, the weights are averaged across the four attention heads.

Fig 4 provides an example of attention weights. It shows the weights assigned between neighboring nodes at each transformer layer in a system with a high Φ value (N=7,Φ=8.036), where all nodes form the major complex. This system was included in the training dataset, and the weights were computed using the model after training was completed. Edge thickness and color intensity represent the magnitude of the attention weights, and for all but the last transformer layer, the weights are averaged across the four attention heads. A common trend is that attention distributions tend to be relatively uniform in the early layers, while deeper layers show more focused patterns, highlighting specific nodes or edges as being more influential for the prediction task. It should be noted, however, that these visualizations do not reveal any clear correspondence to information integration as defined in IIT. In this study, no general or consistent relationship was observed between the learned attention patterns and the IIT framework.

### Estimation in extrapolative setting

The results of the second experiment, where the model was trained and validated on graphs with N=5,6 and tested on graphs with *N* = 7, are presented in Table 2. This setup represents an extrapolative scenario, as the model must generalize to unseen system sizes larger than those used during training. The structure of the table and metrics are consistent with those in the first experiment, and the values represent averages over 100 trials.

**Table 2:** Performance evaluation with training on graphs for N=5,6 and testing on graphs for *N* = 7 (extrapolative). The same comparative experiments as in the previous experiment were conducted. The numbers in parentheses for the proposed method indicate its rank in each column, and results outperforming the proposed method are underlined.

Category	Configuration	Integrated information estimation		Major complex estimation	
		MSE	Correlation	Node-wise accuracy	Graph-wise accuracy
Proposed method		0.8543 (1)	0.6815 (2)	0.8229 (1)	0.4686 (4)
Removed feature	Probability	0.9859	0.6483	0.8130	0.4440
	Parameter *T*	0.9693	0.6307	0.8071	0.4426
	Node degree	0.9621	0.6702	0.8117	0.4477
	Closeness centrality	0.9752	0.6526	0.8036	0.4354
	Betweenness centrality	0.9523	0.6610	0.8107	0.4531
	Clustering coefficient	0.8879	0.6206	0.8141	0.4502
Substitute convolution	GraphConv	1.0821	0.6002	0.7421	0.2974
	GAT	1.1661	0.5638	0.7414	0.3084
Substitute optimizer	Adam	0.8937	0.6778	0.8200	0.4691
	RAdam	0.8980	0.6789	0.8206	0.4710
	AdamW	0.8951	0.6807	0.8198	0.4683
Single task	Φ estimation	0.9964	0.6709	-	-
	Major complex estimation	-	-	0.8228	0.4725
Pooling	Global average	0.8822	0.6726	0.7880	0.3243
	Not subtraction	0.8673	0.6898	0.7951	0.3804

**Fig 5:**
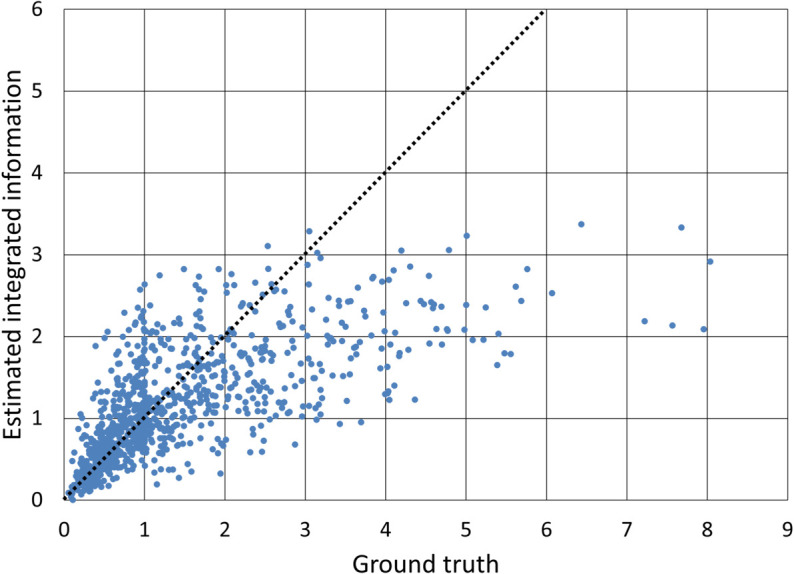
Scatter plot between estimated and true integrated information (extrapolative). The plot shows all 1000 test data corresponding to graphs for *N* = 7 in a specific trial conducted with the proposed method. In this trial, the correlation coefficient was 0.6815, matching the average across all trials.

For integrated information estimation, the performance appears to decline compared to the previous experiment. However, the MSE and the correlation coefficient only for *N* = 7 in the first experiment were 0.7983 and 0.6730, respectively, which are comparable to those in this experiment. Fig 5 shows a scatter plot of estimated versus true integrated information values for a specific trial, illustrating the tendency of the model to underestimate Φ in the high-value range that is not covered by the training data. As high values of integrated information are associated with systems of *N* = 7 in most cases, the integrated information is expected to rise further as the graph size increases beyond *N* = 7; hence, the confidence in integrated information estimates may decrease for such larger graphs.

For the major complex estimation task, both node-wise and graph-wise accuracies also appear to be lower compared to the first experiment. However, considering the test data only for *N* = 7 in the first experiment, the node-wise accuracy was 0.8302, and the graph-wise accuracy was 0.4893, which are nearly consistent with the results here. While the integrated information Φ behaves like an extensive variable and is sensitive to changes in graph size, the membership of each node in the major complex is inherently non-extensive. This suggests that our model may maintain a certain level of reliability in predicting the major complex for larger graphs and exhibit promising extrapolation performance.

In the comparison experiments, we found trends qualitatively similar to those observed in the previous experiment. The removal of one node feature leads to minor declines in major complex estimation accuracy, while integrated information estimation is more sensitive to these changes. The selection of convolution type also has a significant impact, with traditional methods resulting in lower performance than the transformer-based approach. The optimizer selection does not cause a significant difference in performance. Our multi-task approach does not show a clear benefit over single-task estimation, suggesting potential limitations of the proposed method in fully leveraging the theoretical relationship between integrated information and major complex. In addition, global max pooling outperforms global average pooling especially in major complex estimation, because it more effectively captures the most significant feature signals within the graph.

We describe the computational time required by the proposed method. The training process, which used a total of 3000 graphs with *N* = 5 and 6, typically completed within 2 to 3 minutes. Inference of Φ and the major complex for 1000 graphs with *N* = 7 took less than 2 seconds in total. In contrast, computing Φ and the major complex based on the IIT 3.0 framework for a single graph with *N* = 7 required more than 30 minutes on average. The individual computation times varied considerably depending on the number and configuration of edges. Even when the cut-one approximation [14] was applied, the computation time was reduced by only less than 10% on average. These results indicate that our method provides a significant advantage in computational efficiency and is scalable to systems with larger numbers of nodes.

### Scaling behavior of Φ and major complex formation

To predict Φ and the major complex for substantially larger systems, it is important to first evaluate the reliability of such estimates. A direct theoretical analysis of error growth with *N* is intractable, because in IIT 3.0 the value of Φ depends intricately on the interplay between system topology and state-transition probabilities, and the expressive capability of GNNs in this task is not analytically obvious. We therefore investigated whether the trends observed in exact IIT 3.0 calculations for small *N* are preserved in our GNN-based predictions for larger *N*.

**Fig 6:**
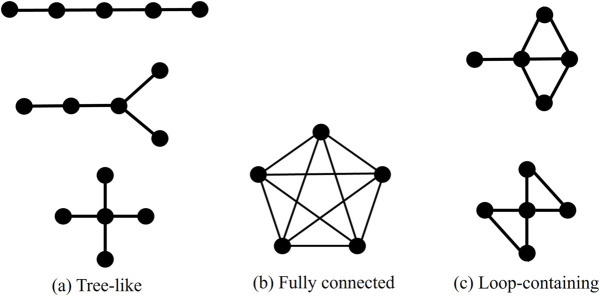
Examples of the three system topologies used in the examination of scaling behavior. These examples show the case of *N* = 5. (a) Tree-like systems contain exactly *N*–1 edges and have no closed loops. (b) Fully connected systems contain all possible edges between the *N* nodes, excluding self-loops. (c) Loop-containing systems have an intermediate number of edges compared with the other two types and include at least one closed loop.

Three types of system topologies, as shown in Fig 6(a), 6(b), 6(c), were considered for the analysis of small *N*:

1. Tree-like systems with *N*–1 undirected edges and no self-loops for *N* ranging from 4 to 10.
2. Fully connected systems for *N* ranging from 4 to 7.
3. Loop-containing systems with an intermediate number of edges. For *N* = 6 and 7, we set 6 and 8 edges, respectively, corresponding to 40% of the edges in the fully connected configuration. For *N* = 4 and 5, we set N+1 edges to ensure that the systems were not too close to the tree-like configuration.

For each *N* and topology, 10 random instances were generated, and Φ and the major complex were computed according to the IIT 3.0 framework.

**Fig 7:**
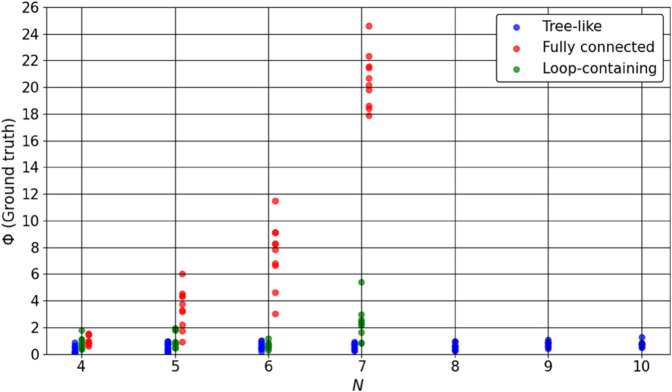
Exact Φ values for tree-like, fully connected, and loop-containing systems with small *N*. For each *N*–topology combination, 10 random systems were generated. Each point corresponds to one instance, plotted with a slight horizontal offset to prevent overlap. For fully connected and loop-containing systems, results are only presented for N≤7 owing to the computational expense of exact IIT 3.0 evaluation.

**Fig 8:**
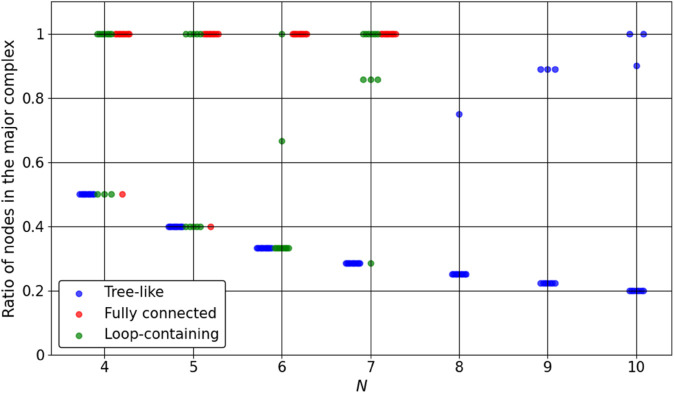
Ratio of nodes included in the major complex for tree-like, fully connected, and loop-containing systems with small *N*. Each point corresponds to one system instance, plotted with a slight horizontal offset to prevent overlap. The ratio is defined as the number of nodes included in the major complex divided by the total number of nodes *N*.

Fig 7 shows the resulting Φ values, and Fig 8 shows the ratio of the number of nodes in the major complex to the total number of nodes in the system. Each plotted point corresponds to one system instance, with a slight horizontal offset to prevent overlap. The results reveal distinct topology-dependent trends:

- Tree-like systems have Φ values ranging from near zero to slightly above 1. The major complex frequently consists of only two nodes, leading to a low ratio in most cases.
- Fully connected systems show a rapid increase in Φ as *N* grows. In many cases, all nodes are included in the major complex.
- Loop-containing systems show intermediate Φ values that increase gradually as *N* grows, and their major complexes often involve a larger number of nodes.

We then applied 100 GNN models, trained only on N=5,6, and 7 in the non-extrapolative setting, to larger systems with N=10,20,…,100. In these large-*N* test systems, we again considered the three topologies: tree-like systems with *N*–1 edges, fully connected systems, and loop-containing systems with 40% of the total number of possible edges. Ten random instances were generated for each *N* and topology. To reduce variability in the predictions across the 100 models, we used an ensemble approach. For each test system, the value of Φ was obtained by averaging the predictions across all models, and a node was considered to be included in the major complex if at least 60% of the models predicted its inclusion.

**Fig 9:**
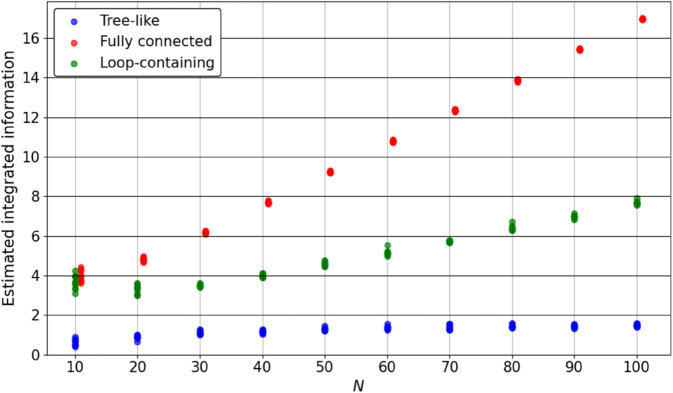
Estimated integrated information Φ for tree-like, fully connected, and loop-containing systems with larger *N*. For each *N*–topology combination, 10 randomly generated systems were evaluated, and each point corresponds to one system instance. Tree-like systems show Φ values that remain within a narrow range across *N*. For fully connected and loop-containing systems, Φ tends to increase with *N*, but the values are considerably smaller than those implied by the exact results for small *N* (see Fig 7).

**Fig 10:**
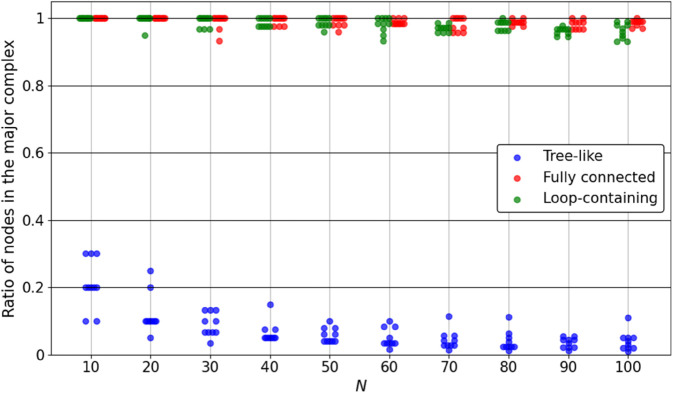
Estimated ratio of major complex size for tree-like, fully connected, and loop-containing systems with larger *N*. Each point represents one system instance, plotted with a slight horizontal offset to prevent overlap. Tree-like systems generally show low ratios, whereas fully connected and loop-containing systems tend to include most or all nodes in the major complex.

Fig 9 and Fig 10 show the estimated Φ values and major complex ratios, respectively. Each plotted point corresponds to one system instance, with a slight horizontal offset for clarity. Most of the qualitative trends observed in the exact computation for small *N* are preserved:

- Tree-like systems maintain low Φ and small major complexes.
- Fully connected systems show increasing Φ with *N*. In many cases, all nodes are included in the major complex.
- Loop-containing systems exhibit intermediate Φ values that increase with *N*, and major complexes spanning almost the entire system. The major complex size appears to follow the trend implied by exact values for small *N*.

However, for both fully connected and loop-containing systems, the estimated Φ values are lower than those implied by the exact results for small *N*.

These results suggest that, while our proposed method cannot accurately estimate Φ values for large *N*, it may still be useful for comparing different systems in terms of Φ or for identifying topology-dependent trends. Consequently, our method can be applied to reveal qualitative features of information integration and major complex formation in large-scale systems where exact IIT 3.0 computation is not feasible.

### Qualitative analysis of split-brain-like systems

Building on the results of the previous section, we perform a qualitative analysis of a simplified yet structurally meaningful system that mimics a specific brain condition known as the “split brain.” Our system employs only 100 nodes, far fewer than the number of neurons in an actual brain. Nevertheless, this system is designed to capture the structural separation between the two hemispheres. By applying our GNN-based estimation models to this system, we examine how integrated information Φ and the major complex change as inter-hemispheric connectivity varies. This approach enables the investigation of split-brain–like disconnections within a tractable computational setting, and it allows us to explore their potential implications for understanding brain network organization.

Each test system comprised two subsystems: for the first 50 nodes, edges were randomly generated between pairs of nodes with a probability of 0.6, while the remaining 50 nodes were connected with a probability of 0.4. An edge connecting nodes between the two subsystems was set with probability *p*_*e*_ ranging from 0 to 0.4. For all edges, their strengths were independently sampled from a standard normal distribution. In the case of *p*_*e*_ = 0, the two subsystems are completely separate as in Fig 2, resembling a split-brain scenario. As the value of *p*_*e*_ increases, inter-subsystem connections are introduced, resulting in progressively greater integration between the subsystems. Specifically, we were interested in observing how the system transitions from the local integration to the global integration.

Fifty large-scale test systems were generated for each value of *p*_*e*_. From the first experiment in the non-extrapolative setting, we obtained 100 estimation models trained on smaller systems with N=5,6, and 7. These models were then applied to the large-scale systems to predict the integrated information and identify the major complex. To enhance reliability, considering the variability in individual model predictions especially for extrapolative conditions, we averaged the estimated values of Φ across the 100 models and determined that a node belonged to the major complex if at least 60% of these models supported its inclusion.

**Fig 11:**
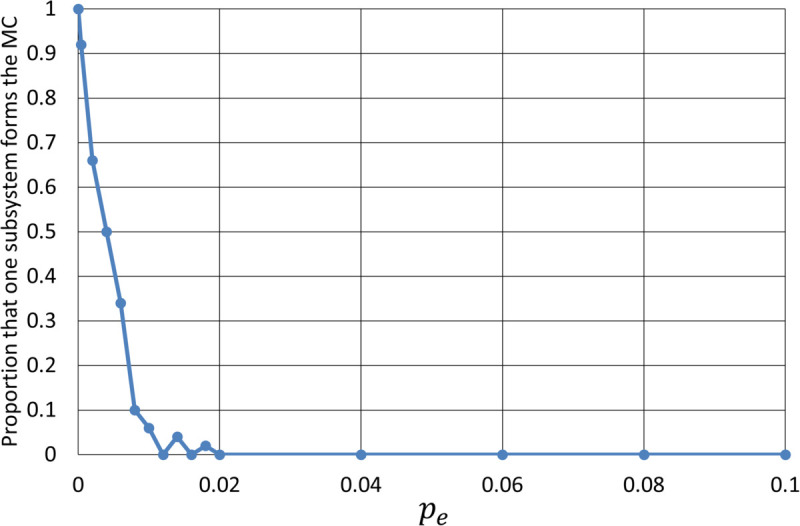
Proportion of cases out of 50 test systems where the single subsystem (the first 50 nodes) forms the major complex (MC) as a function of inter-subsystem edge probability $p_{e}$. At *p*_*e*_ = 0, the major complex consists of only the first 50 nodes. As *p*_*e*_ increases, this rate rapidly declines.

Fig 11 shows the percentage of cases in which the subsystem of the first 50 nodes forms the major complex out of the 50 test systems by varying the value of *p*_*e*_. In all trials with *p*_*e*_ = 0, the major complex is composed of the first 50 nodes with denser internal connectivity. Identifying a single subsystem as the major complex is facilitated by the data augmentation techniques described in the Methods section and the operation of subtracting the max-pooled vector from the feature of each node. As *p*_*e*_ increases, this proportion of “local integration” declines rapidly, and when *p*_*e*_ reaches around 0.01 to 0.02, the major complex spans both subsystems with nearly 100% certainty.

**Fig 12:**
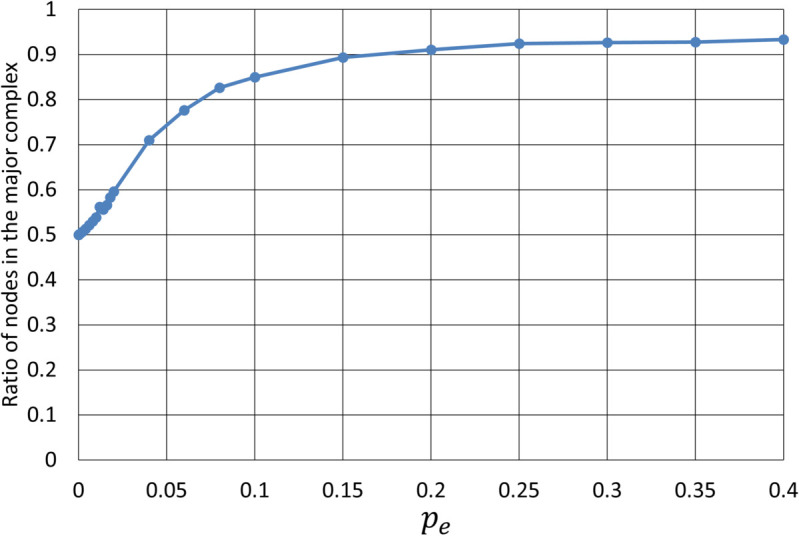
Ratio of nodes included in the major complex out of 100 total nodes as a function of $p_{e}$. The values of ratios were averaged over 50 test systems. The ratio is 0.5 at *p*_*e*_ = 0, and it gradually increases with *p*_*e*_, eventually reaching around 0.93.

Fig 12 shows the ratio of nodes included in the major complex out of the total 100 nodes as the value of *p*_*e*_ varies. This ratio was averaged over 50 test systems. At *p*_*e*_ = 0, the major complex is formed by all nodes in the first subsystem across all test systems, resulting in a ratio of 0.5. As *p*_*e*_ increases toward 0.02, the local integration disappears rapidly; by contrast, this ratio progresses more slowly, reaching only around 0.6. As *p*_*e*_ increases further, the ratio rises steadily and eventually reaches approximately 0.93. This indicates that a larger fraction of the system contributes to the formation of the major complex, which can be regarded as “global integration.” This behavior may be akin to a functional shift from localized processing to a more distributed and unified form of information integration, as observed in neural circuits of the brain.

**Fig 13:**
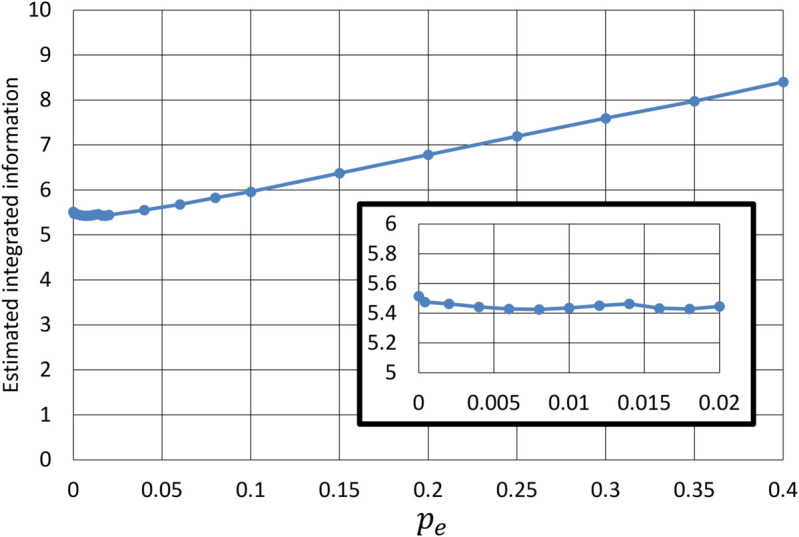
Estimated integrated information Φ as a function of $p_{e}$. The values were averaged over 50 test systems and 100 estimation models. For small *p*_*e*_, the values of Φ remain nearly constant as illustrated in the interpolated diagram. It begins to increase as the major complex spans both subsystems, reflecting higher integration with increased connectivity.

Fig 13 shows the estimated integrated information Φ averaged over 50 test systems and 100 models as a function of *p*_*e*_. Based on the scaling behavior, the estimated values of Φ are not highly reliable; however, relative changes in Φ can provide insights into significant trends. When *p*_*e*_ is sufficiently small $\left(p_{e}≲0.02\right)$ such that test systems resemble a split brain, the values of Φ remain approximately constant. As *p*_*e*_ increases and the major complex expands to include more nodes from both subsystems, the value of Φ grows correspondingly, indicating that enhanced interconnections and interactions between subsystems correlates with higher integrated information. As with the increase in the major complex size discussed above, such growth in the value of Φ might correspond to a transition from localized to cohesive and unified processing in the brain.

These results illustrate the capacity of our model to differentiate between local and global integration in the system. For lower *p*_*e*_ values, the system exhibits characteristics similar to a split brain, where the major complex is restricted to one of the subsystems. As *p*_*e*_ increases and the inter-subsystem connectivity increases, the model begins identifying a unified major complex that spans both subsystems, corresponding to a globally integrated configuration. This highlights the potential of the GNN model to effectively make predictions based on large-scale connectivity patterns and to explore how system integration relates to neural configurations, although further validation across different datasets and scenarios is required.

## Discussion

This study explored the applicability of GNNs for estimating integrated information and major complex as defined by IIT 3.0. Our GNN models could capture qualitative trends in both Φ and the major complex through their estimations in large systems. However, the multi-task architecture showed clear limitations, as our model was not able to fully exploit the intricate relationships between the integrated information and the major complex. These limitations highlight the need for refining the model architecture, which we discuss below.

One potential enhancement involves expanding the predictive scope of the model to include additional variables (see S1 Text), such as the “mechanism-level” integrated information φ (small phi) and the integrated information of unidirectionally-partitioned subsystems. Although these variables span different computational stages within the IIT 3.0 framework, they can be represented within a heterogeneous graph [42,43]. For example, consider “mechanisms” in IIT 3.0, which are defined as subsets of system nodes and are characterized by their own φ values. One could extend the graph by introducing “mechanism nodes,” distinct from the original system nodes, and by connecting them to their constituent system nodes through edges of a different type. This results in a heterogeneous graph that includes not only the original system structure but also mechanism-level nodes and edges. Training a GNN on this graph allows the model to learn multi-dimensional embeddings for mechanism nodes from their constituent system nodes. Using these embeddings, a subsequent regression layer can then predict integrated information φ of each mechanism. Since embeddings of mechanism nodes propagate back into their constituent system nodes through message passing, incorporating this mechanism-level information may in turn improve the accuracy of system-level predictions for Φ and the major complex. The total number of mechanisms grows exponentially with *N*, so not all mechanism nodes can be constructed for large systems. Therefore, in practice, a heterogeneous graph for test systems can be constructed by randomly generating mechanism nodes and their associated edges.

One potential improvement in the training strategy is to incorporate contrastive learning. This technique assigns latent representations (embedding) to input data such that semantically similar data have close representations, while dissimilar data have distinct ones [44–46]. This is achieved by introducing a contrastive term into the loss function to encourage this behavior. In graph-based contrastive learning, similarity is typically defined by node features or graph topology. However, in our problem, graphs with different topologies can yield similar integrated information Φ; thus, defining similarity only by topology is inappropriate. Therefore, we propose to define similarity on the basis of Φ or major complex composition, in analogy to the use of class labels in supervised contrastive learning [46]. In our model, the graph representation is obtained by applying global pooling to the output of the fourth transformer convolution layer. A contrastive term based on this embedding can be added to the overall loss function, where graphs with similar values of integrated information Φ or similar major complex composition are treated as positive pairs, while the others are treated as negative pairs. This contrastive loss may improve the prediction accuracy of our GNN model.

The proposed model faced challenges in accurately estimating integrated information as the number of nodes increased, particularly when extrapolating to larger systems. Underestimation in the region of higher values suggests that current training process might not sufficiently capture the scaling factors relative to the number of nodes. This issue could be addressed by treating the scaling factors as learnable parameters. One promising solution is to adopt an iterative learning approach consisting of two steps: (1) training the network weights on smaller graphs such as *N* = 5 or *N* = 6 with the scaling factors fixed, and (2) training only the scaling factors on larger graphs such as *N* = 7 with the network weights fixed. By repeating these steps, the model may progressively enhance its extrapolative estimation ability.

Despite the aforementioned limitations of current models, the approach developed in this study can be applied to IIT 4.0, the latest version of the theory. This is because the main objective of IIT 4.0 remains the same: estimating integrated information Φ and identifying the associated complex based on nodes, edges, and transition probabilities of a system. Furthermore, the advanced computational process in IIT 4.0 introduces the concept of “relations,” which specify connections between groups of nodes. These relations could also be effectively represented by a heterogeneous graph. In particular, “relation nodes” could be added as a new node type, with edges connecting each relation node to the original system nodes that constitute the relation. This could help GNNs capture higher-order interactions and improve the prediction of Φ and its associated complex.

From the formulation of the transformer convolution (Eqs (2)–(4)), learnable parameters in the model are contained in the weight matrices $W_{q},W_{k},W_{v},W_{e}$ and $W_{0}$, which are shared across all nodes and are not directly influenced by the number of nodes *N*. By contrast, it is necessary to compute the attention coefficients $\alpha_{ij}$ (Eq (3)) for every edge in the graph. Additionally, deriving centrality-related node features becomes computationally expensive as *N* grows, since such computations often have a time complexity greater than *O*(*N*). Taken together, if the number of connections per node remains *O*(1) and the centrality features are substituted with computationally efficient alternatives, the computational cost can be substantially reduced. Under these conditions, the proposed method should remain feasible even for much larger *N* than those considered here within the limits of available computational resources, particularly memory capacity. This suggests that the proposed method may be applicable to real-world neural systems. For example, connectome-level brain networks obtained from human or animal studies [47,48] could serve as valuable testbeds for examining integrated information and major complexes in large biological systems.

In conclusion, our GNN-based model provides a promising approach for approximating IIT calculations, particularly for large systems. Further research should focus on refining the model architecture, exploring scaling strategies, and enhancing the multi-task framework to improve prediction accuracy. Ultimately, developing such a tool will provide a deeper understanding of consciousness and other emergent phenomena. It may also offer crucial insights for bridging computational models with neuroscience.

## Supporting information

S1 Text Nested optimization framework in IIT 3.0. (PDF)
