# Supplementary material for: Graph neural networks for integrated information and major complex estimation
Source: PLoS One. 2025 Nov 7;20(11):e0335966. doi: 10.1371/journal.pone.0335966 (PMC12594358; doi:10.1371/journal.pone.0335966)
Supplement: S1 Text — (PDF) [file pone.0335966.s001.pdf]

Graph neural networks for integrated information and major complex estimation

Supporting information (S1 Text)

Tadaaki Hosaka[1]

1 School of Science and Technology, Meiji University, Kanagawa, Japan

* hosaka.tadaaki@gmail.com

Nested optimization framework in IIT 3.0

This appendix outlines the nested optimization framework for calculating integrated information at the mechanism level and system level. The purpose here is to illustrate the computational complexity of IIT 3.0, without addressing the core principles, such as its underlying axioms and postulates which are central to the foundational philosophy of IIT.

Mechanism-level integrated information

A system is represented by a graph consisting of nodes and edges. Assume that for this system, the state of each node, the weight of each edge, and the transition probabilities between states are known. Let S denote a subsystem within this system, and let M be a set of nodes within S; this set is referred to as the mechanism. To evaluate the integrated information of this mechanism, we proceed with the following steps:

1. **Derive cause and effect distributions**: For the mechanism M, we define an effect purview P_+ and a cause purview P_- as a set of nodes. Based on the transition probability matrix of the system, we compute the conditional probability distribution $p(P_\pm|M;S)$ for these purviews.

2. **Partition the system and measure the distance between distributions**: Then, we consider a partitioned version of the system, where some causal connections are removed to divide the system into parts, yielding a distribution $p^{\mathrm{cut}}(P_\pm|M;S)$ for the partitioned system. We calculate the distance D between the distributions of the non-partitioned and partitioned systems using the earth mover's distance as a metric.

3. **Optimize with respect to partitions and purviews**: For each purview P_+ and P_-, we seek the partition that minimizes the distance D. Then, we select the purviews P_+ and P_- that maximize this minimized distance, resulting in the following expressions for the cause and effect integrated information:

$$\varphi_\pm(M;S) = \max_{P_\pm} \left[\min_{\mathrm{cut}} D\left(p(P_\pm|M;S), p^{\mathrm{cut}}(P_\pm|M;S) \right) \right].$$

   Finally, the integrated information for the mechanism M is defined as follows:

$$\varphi(M;S) = \min\left\{ \varphi_+(M;S), \varphi_-(M;S) \right\}.$$

The aforementioned process is repeated for every possible mechanism M in subsystem S, forming a collection called the conceptual structure $\mathcal{C}(S)$:

$$\mathcal{C}(S) = \left\{\varphi(M;S), p(P_+^*|M;S), p(P_-^*|M;S)\right\}_M ,$$

where mechanisms are restricted to those with $\varphi(M;S) > 0$, and $*$ indicates the optimal purviews.

System-level integrated information

To evaluate the integrated information at the system level, we proceed with the following steps:

1. **Cut the system unidirectionally and measure the distance between conceptual structures**: For a given subsystem S, we apply a unidirectional cut that removes some causal connections in one direction, resulting in an altered version of the subsystem with a conceptual structure denoted by $\mathcal{C}^{\text{unicut}}(S)$. Then, we compute the distance D' between the conceptual structure of the original subsystem $\mathcal{C}(S)$ and that of the unidirectionally cut subsystem $\mathcal{C}^{\text{unicut}}(S)$ using an extended version of the earth mover's distance.

2. **Optimize with respect to cuts and subsystems**: For each subsystem S, we identify the unidirectional cut that minimizes the distance D' between $\mathcal{C}(S)$ and $\mathcal{C}^{\text{unicut}}(S)$, defining the system-level integrated information $\Phi(S)$ as follows:

   $$\Phi(S) = \min_{\text{unicut}} D'\left(\mathcal{C}(S), \mathcal{C}^{\text{unicut}}(S)\right).$$

   Then, we find the subsystem S that maximizes $\Phi(S)$ across all subsystems, and define the major complex as follows:

   $$\Phi = \max_S \Phi(S),$$

   $$\text{Major complex} = \operatorname*{argmax}_S \Phi(S).$$

   The major complex represents the part of the system contributing most significantly to overall consciousness.

Each optimization concerning partitions, purviews, unidirectional cuts, and subsystems, as well as investigating all mechanisms, is computationally challenging, as all combinations must be evaluated. Furthermore, because these optimizations are nested, the computational complexity grows at a super-exponential rate, making it practically infeasible to perform these calculations within realistic time constraints except for extremely small-sized systems.
